# Impaired Perception and Neural Processing of Rules in Developmental Dyslexia

**DOI:** 10.1177/0022219420988004

**Published:** 2021-01-21

**Authors:** Paula Virtala, Eino Partanen, Teija Kujala

**Affiliations:** 1 University of Helsinki, Finland

**Keywords:** auditory processing, dyslexia, cognitive neuroscience

## Abstract

Rules and regularities of language are typically processed in an implicit and effortless way in the human brain. Individuals with developmental dyslexia have problems in implicit learning of regularities in sequential stimuli, but the neural basis of this deficit has not been studied. This study investigated extraction and utilization of a complex auditory rule at neural and perceptual levels in 18 adults with dyslexia and 20 typical readers. Mismatch negativity (MMN) and P3a responses to rule violations in speech stimuli, reflecting change detection and attention switch, respectively, were recorded with electroencephalogram. Both groups reported no or little explicit awareness of the rule, suggesting implicit processing. People with dyslexia showed deficient extraction of the rule evidenced by diminished MMNs estimated to originate particularly from the left perisylvian region. The group difference persisted in the attentive condition after the participants were told about the rule, and behavioral detection of the rule violations was poor in people with dyslexia, possibly suggesting difficulties also in utilizing explicit information of the rule. Based on these results, the speech processing difficulties in dyslexia extend beyond phoneme discrimination and basic auditory feature extraction. Challenges in implicit extraction and effortless adoption of complex auditory rules may be central to language learning difficulties in dyslexia.

Language has a profound position in human neurocognition, and the auditory modality is central in its acquisition and processing. During early development, infants learn the native language phonemes by hearing them (Kuhl, 2004) and a wide neural network for language is formed (Friederici, 2012; Friederici & Gierhan, 2013; Hickok & Poeppel, 2004; Price, 2010). Auditory processing is compromised in neurodevelopmental language disorders such as the reading deficit developmental dyslexia or developmental language disorder (DLD; reviews: Hämäläinen et al., 2013; Kujala & Leminen, 2017; Schulte-Körne & Bruder, 2010). For example, people with dyslexia have difficulties in discriminating changes in basic auditory features as well as speech sounds (Hämäläinen et al., 2013; Schulte-Körne & Bruder, 2010). Dyslexia is primarily thought to be a phonological processing deficit (Giraud & Ramus, 2013; Vellutino et al., 2004). However, language is much more than phonemes: It contains complex rules and regularities that define how the language units go together.

Acquiring one’s native language, including its rules and regularities, occurs implicitly during early development. Learning is considered *implicit* when it is unintentional and the resulting knowledge is difficult to express verbally (see, e.g., Cleeremans et al., 1998). The role of implicit learning in dyslexia has received interest during the recent decades (for a meta-analysis, see Lum et al., 2013). It has been proposed that problems in implicit and procedural memory (but not in explicit memory) would be characteristic of learning deficits like dyslexia (Pennington et al., 2019; see also Krishnan et al., 2016; Nicolson & Fawcett, 2007; Ullman & Pierpont, 2005), and that the difficulties in implicit learning of people with dyslexia are central in their problems in acquiring a fluent reading skill (e.g., Pavlidou & Williams, 2014; Sperling et al., 2004). Implicit learning is often studied with the Serial Reaction Time Test (SRTT; Lum et al., 2013; Nissen & Bullemer, 1987) and the Artificial Grammar Learning (AGL) task (Pothos, 2007; Reber, 1989). Both of these behavioral tasks present an underlying regularity in a series of visual stimuli, with SRTT even including a motor skill–learning component.

In the auditory modality, studies on implicit learning, particularly those tapping brain processes, are scarce despite the importance of auditory processing for language learning and its deficits as discussed above. In two behavioral experiments, Gabay et al. (Gabay et al., 2015; Gabay & Holt, 2015) showed that incidental learning of auditory categories and statistical learning of auditory regularities were impaired in people with dyslexia, who were poorer than typical readers in detecting transitional probabilities in speech and nonspeech sounds after passive exposure to them (Gabay et al., 2015) and in learning novel auditory categories during a video game (Gabay & Holt, 2015). Based on their results, the authors suggested that problems in implicit (or procedural) learning may result in poor acquisition of phoneme categories during early development, which is understood to be a central problem in dyslexia (e.g., Eden et al., 2016; Peterson & Pennington, 2015). Thus, the implicit learning deficit might be one of the underlying causes for the phonological deficits in dyslexia.

A well-established method for studying neural auditory processing in neurodevelopmental disorders is to record event-related potentials (ERPs) of the electroencephalogram (EEG). In a recent ERP study, passive exposure to a single novel word resulted in an enhancement in the ERP waveform in typical readers but not in children with dyslexia (Kimppa et al., 2018; see also Perrachione et al., 2016). The result might reflect impaired rapid (implicit) learning of the novel word form during the experiment for children with dyslexia in line with the behavioral results described above (Gabay et al., 2015; Gabay & Holt, 2015). However, the studied process (exposure to a single novel word) was quite different from the behavioral studies presenting regularities in a series of visual or auditory stimuli. As far as we know, only a few studies have utilized auditory ERPs to study such processing in dyslexia (Bonte et al., 2007; Cantiani et al., 2013, 2015; Sabisch et al., 2006). In these ERP studies, people with dyslexia have displayed abnormal processing of rules that should have been well established during language development, like phonotactic probabilities (Bonte et al., 2007) and morphosyntactic violations (Cantiani et al., 2013, 2015; Sabisch et al., 2006) in their native language. For example, Cantiani et al. (2015) reported an N400 enhancement in people with dyslexia in response to morphosyntactic violations and interpreted the finding as a compensatory mechanism for the “difficulties in constructing implicit rules” of people with dyslexia. However, as Gabay et al. (2015) pointed out, presenting a rule belonging to the participants’ native language can lead to confounding effects of prior language or speech processing skills. This can be avoided by introducing a novel stimulus or artificial rule in the experiment.

A particularly feasible tool for studying implicit extraction of auditory rules with the ERPs is mismatch negativity (MMN), which is elicited by violations of regularities in the auditory input irrespective of the direction of the individual’s attention (for a review, see Paavilainen, 2013). A so-called abstract-MMN was originally recorded with a paradigm in which sound pairs with a rising pitch (first sound lower than the second) were presented at various frequency levels and, occasionally, a deviant sound pair with a falling pitch was presented (i.e., the sound order changes—first sound higher than the second; Saarinen et al., 1992; see also Paavilainen et al., 1999; for review, see Paavilainen, 2013). MMN elicitation in such a context requires implicit extraction of the underlying regularity. The MMN responses to these rule violations were even found when the participants could not explicitly describe the violation afterward (van Zuijen et al., 2006). However, P3a responses, reflecting attention switch to stimulus changes (e.g., Wetzel & Schröger, 2014), were only evident in participants who could verbally describe the rule and detect the rule violations in an attentive listening condition (van Zuijen et al., 2006). In contrast with these results, a recent study showed that both MMN and P3a responses were elicited by rule violations in a speech sound context, in both ignore and attentive listening conditions, even in the absence of explicit awareness of the rule (Virtala et al., 2018; for similar P3a results with nonspeech stimuli, see Paavilainen et al., 2007). Thus, whether the P3a is also elicited in truly implicit processing settings still remains debatable.

The aim of this study was to investigate whether implicit auditory rule extraction at the neural and behavioral levels is compromised in developmental dyslexia. To our knowledge, implicit processing has not been studied previously in dyslexia or other neurodevelopmental disorders with the abstract-MMN despite its feasibility for tapping implicit rule extraction. By recording pre-attentive ERPs instead of employing a behavioral task, it is possible to tap lower level cognitive processes that are less likely to be affected by group differences in, for example, attention span or motivational factors. To this end, a classical abstract-MMN paradigm was employed in a speech sound context. We recorded MMN and P3a responses to the rule violations in ignore and attentive listening conditions and assessed their detection with behavioral tests. Explicit awareness of the rule was controlled for by asking the participants to describe it verbally and then informing all the participants about the rule before the attentive/explicit listening condition, including the behavioral detection task. This was done to ensure that all participants had the same amount of explicit information about the stimuli and to compare how people with dyslexia versus typical readers could adopt and utilize this explicit information.

The research questions were as follows:

**Research Question 1 (RQ1):** Does dyslexia compromise auditory rule extraction at the neural and behavioral level in the ignore/implicit and attentive/explicit conditions?
**Research Question 2 (RQ2):** Which cortical sources demonstrate group differences in auditory rule extraction?
**Research Question 3 (RQ3):** Is auditory rule extraction associated with reading and related skills?

Based on previous evidence of the deficient processing of auditory regularities by people with dyslexia (Bonte et al., 2007; Cantiani et al., 2013, 2015; Gabay et al., 2015; Gabay & Holt, 2015; Kujala et al., 2000; Sabisch et al., 2006), we hypothesized that they would demonstrate diminished or absent MMN/N2bs, and possibly also P3as, as well as poor behavioral detection of the rule violations. Should the group difference be evident only in the ignore/implicit condition, it would offer neural evidence for the proposed implicit but not explicit learning deficit in dyslexia (e.g., Lum et al., 2013). However, should the group differences persist in conditions with explicit stimulus processing, it could suggest difficulties in utilizing the explicit information of the auditory rule in dyslexia.

The neural sources of auditory rule extraction were expected to be weaker in people with dyslexia approximately in the left temporal-frontal regions where the abstract-MMN to language stimuli should originate from (Paavilainen, 2013). As implicit learning difficulties are suggested to be causally related to the reading deficit in dyslexia (e.g., Pavlidou & Williams, 2014; Sperling et al., 2004), and as a previous study on implicit novel word form learning in children with dyslexia demonstrated associations to reading skills (Kimppa et al., 2018), we also expected associations between more efficient auditory rule extraction (larger MMN/N2b responses, possibly particularly in the ignore/implicit condition) and better reading and related skills.

## Method

### Participants

Thirty-nine participants, recruited through social media and a website, were included in the analyses (18 people with dyslexia and 20 control group members); one participant reported having absolute pitch and was excluded because absolute pitch may affect auditory ERPs in a paradigm including pitch changes (e.g., Rogenmoser et al., 2015). All participants completed a neuropsychological test battery as described in Table 1. A participant was considered to have dyslexia if their speed or accuracy was 1*SD* below the expected level in at least two out of three reading tests (Nevala et al., 2006: word list and pseudoword list reading, reading a narrative text; control data by Laasonen et al., 2010). All people with dyslexia had to have symptoms dating back to childhood as found in an interview or in the Adult Reading History Questionnaire (ARHQ; Lefly & Pennington, 2000). No present or past reading problems were reported by the controls. Participants in both groups reported to be right-handed, native monolingual speakers of Finnish, having normal hearing and vision normal or corrected-to-normal, no problems in basic motor functions, and no neurological or psychiatric diseases, or (other) learning-, language-, or attention-related impairments (these were additionally screened via the Adult ADHD Self-Report Scale [ASRS-V1.1] Symptom Checklist, Kessler et al., 2005). The groups were balanced in relevant background factors: age 32.5 (*SD* = 8.9) years in people with dyslexia versus age 29.5 (*SD* = 7.9) years in control group, independent samples *t*-test *p* > .20; gender distribution, nine of 18 males in dyslexia group versus 10 of 20 males in control group, chi-square test *p* > .20; duration of education, 15.0 (*SD* = 2.6) years in people with dyslexia versus 16.3 (*SD* = 2.7) years in control group, independent samples *t*-test *p* = .143.

**Table 1.** Neuropsychological Test Battery and Test Scores (*SD*).

Subtest/index	Controls^a^	Dyslexics	Reference
PIQ,^b^ WAIS-III	120.2 (10.1)	112.9 (11.9)	Wechsler (2005)
VIQ,^b^ WAIS-III	112.3 (8.7)	103.8 (7.5)	Wechsler (2005)
Digit span,^c^ WMS-III	12.2 (3.0)	9.3 (1.7)	Wechsler (2008)
Visual series, WMS-III	9.3 (2.4)	10.2 (3.3)	Wechsler (2008)
Word lists I, WMS-III	10.6 (3.0)	8.8 (2.3)	Wechsler (2008)
Word lists II, WMS-III	11.3 (3.9)	10.1 (2.4)	Wechsler (2008)
Nonword span, length^c^	4.4 (0.6)	3.9 (0.3)	Laasonen et al. (2002)
RAS, speed in second trial in s^c^	26.5 (6.0)	32.7 (5.2)	Wolf (1986)
Word list reading, time^c,d^	19.9 (3.2)	27.7 (6.3)	Nevala et al. (2006)
Nonword list reading, time^c,d^	41.4 (10.0)	64.1 (16.3)	Nevala et al. (2006)
Text reading, amount of words^c,d^	431.1 (40.2)	334.3 (52.6)	Nevala et al. (2006)
Pig Latin^c^	13.3 (3.5)	10.2 (4.6)	Nevala et al. (2006)

*Note*. Standardized scores are reported. PIQ = performance intelligence quotient; VIQ = verbal intelligence quotient; WAIS-III = Wechsler Adult Intelligence Scale–Third Edition (Wechsler, 2005); WMS-III = Wechsler Memory Scale–Third Edition (Wechsler, 2008); RAS = rapid alternating stimulus.

a Neuropsychological test data were missing from three control participants due to scheduling issues. Therefore, no group comparisons were conducted on the test scores, and they were used only for correlation analyses. ^b^Performance IQ (PIQ) and verbal IQ (VIQ) were calculated from Matrix Reasoning and Block Design and Similarities and Vocabulary WAIS-III (Wechsler, 2005), respectively. ^c^Included in correlational analyses. ^d^In the reading subtests also accuracy was measured but it included very little variation and therefore only speed was included in correlation analyses.

Written informed consent to participate in the study was obtained from all participants and a compensation (vouchers for cultural or exercise activities) was provided after the study. The University of Helsinki Review Board in Humanities and Social and Behavioral Sciences granted ethical approval for the study. Partly overlapping data of 10 control group participants of this study were reported in Virtala et al. (2018) and different data of the participant sample of this study were reported in Virtala et al. (2020).

### Experimental Stimuli and Paradigms

The experimental stimuli, paradigms, procedure, and EEG recording have been described in detail in Virtala et al. (2018). The stimuli were pairs of naturally uttered phonemes /i/-/i/ and /i/-/æ/, edited (Praat 5.4.01, Boersma & Weenink, 2013; Adobe Audition CS6 5.0. Build 708, Adobe Systems Inc., California, USA) so that the sound intensity was root-mean-square normalized between phonemes, and the phoneme duration was cut to 230 ms with a smooth ending at 190 to 230 ms. The phonemes had a natural F0-level of 206.8 Hz and they were transposed to seven additional F0-levels (174.3, 184.6, 195.4, 217.8, 229.7, 242.5, and 256.2 Hz). Phoneme pairs had a duration of 530 ms (70-ms silent gap in between). Within the phoneme pairs, frequency difference between the phonemes was always more than one frequency level, with /æ/ always having a higher frequency than /i/ in /i/-/æ/ pairs, resulting in 42 different /i/-/i/ pairs (21 rising and 21 falling in frequency) and 21 different /i/-/æ/ pairs.

**Figure 1.**
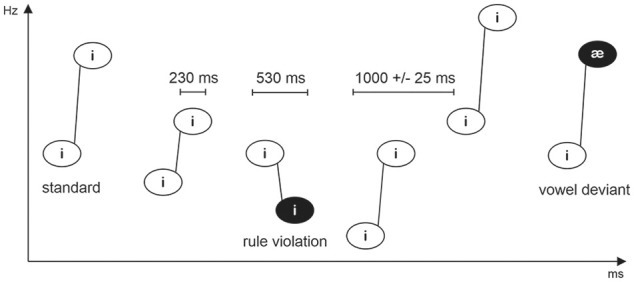
Experimental paradigm used in the study. *Source*. Adapted from Virtala et al. (2018).

The experimental paradigm is described in Figure 1. The /i/-/i/ pairs rising in frequency acted as the standard (probability 80%), and /i/-/i/ pairs falling in frequency (rule violation, 10%) and /i/-/æ/ pairs (vowel deviant, 10%) acted as deviants. The data recorded for the vowel deviants have been reported elsewhere (Virtala et al., 2020). The phoneme pairs were presented pseudo-randomly, so that the sequence started with at least seven standards and at least one standard preceded every deviant. To reduce phase-locked brain activity due to regularly repeating stimuli, the onset-to-onset interval between tone pairs included a ±25-ms jitter in 10-ms steps (thus being 975, 985, 995, 1,005, 1,015, or 1,025 ms).

### Experimental Procedure

There were four stimulus sequences presented with the Presentation software V 17.2 (NeuroBehavioral Systems Inc., CA, USA) binaurally through headphones (Sony Dynamic Stereo Headphones, MDR-7506), while the participant was seated comfortably in a soundproof, electrically shielded room (see Table 2). Stimulus intensity was approximately 65 dB SPL(a). The Cedrus RB844 response pad (Cedrus Corporation, CA, USA) was used to record the behavioral responses during AttendVowel and AttendRule.

**Table 2.** Experimental Sequences and Protocol.

Sequence	EEG	Instructions (I), correct answers (A), and queries (Q) to the participants
Ignore	Yes	I: “Your task is to focus on the silent movie and ignore the presented sound stream.”
		Q: “The sound stream you heard varies constantly, but occasionally, two types of changes occur in it. Can you name these changes in the sound stream?”
Familiarization	No	I: “Next, focus on listening to the sound stream and think what changes occur in it. Push the response box button always immediately after hearing a change.”
		Q: “Can you now tell what kind of changes occur in the sound stream?” A: “In the stream of speech sounds, there are vowel changes (from /i/ to /æ/) and rule violations as follows: when in most of the vowel pairs, pitch rises so that first sound is lower than the next sound, in some vowel pairs it falls, so that first sound is higher than the next sound.”
AttendVowel	Yes	I: “Again, focus on listening to the sound stream. Push the response box button always immediately after hearing the vowel change. Ignore the rule violation.”
AttendRule	Yes	I: “Again, focus on listening to the sound stream. Push the response box button always immediately after hearing the rule violation. Ignore the vowel change.”

*Source*. Adapted from Virtala et al. (2018).

*Note*. Instructions (I) were always given before the sequence, and queries (Q) and correct answers (A) after it. AttendVowel and AttendRule sequences were presented in a counterbalanced order between participants. EEG = electroencephalogram.

The experiment started with the ignore EEG recording condition (sequence Ignore, 21 min, 1,260 stimuli, 126 × 2 deviants) in which the participant watched a subtitled movie without sounds and was instructed to ignore any sounds. Afterward, the participant was asked to describe the deviants in the preceding sequence. Next, the participant was told that there were two types of changes and was asked to detect both deviants by pressing a button during a familiarization phase (Familiarization, 90 s, 90 stimuli, 9 × 2 deviants). After Familiarization, the participant was again asked to describe the deviants in the preceding sequence, and then was given explicit information about the rule (and the vowel deviant). In an attentive condition in two separate sequences, the participant was then instructed to detect the rule violations (AttendRule, ~10 min, 630 stimuli, 63 × 2 deviants) and the vowel deviants (AttendVowel, ~10 min, 630 stimuli, 63 × 2 deviants) during EEG recording. The presentation order of these two detection tasks was counterbalanced between participants. The participants were told to avoid movements and excessive blinking during the EEG recording. No EEG was recorded during Familiarization.

### EEG Recording and Analysis

The EEG was recorded with a sampling rate of 512 Hz with the BioSemi amplifier and 64-active-electrode cap (electrode placing according to the international 10–20 system, online referenced to the CMS, Biosemi ActiveTwo mk2, BioSemi B. V., Amsterdam, The Netherlands). Additional external Ag/AgCl electrodes were attached on left and right mastoids, nose, and below and to the right of the right eye (vertical and horizontal electrooculogram).

EEG was preprocessed with BESA Research 6.0 Software (BESA GmbH, Gräfelfing, Germany). It was re-referenced (average of the two mastoid electrodes) and filtered at 1 to 30 Hz (slope 12 dB/oct., zero phase), and electrodes with high-frequency noise were either omitted (peripheral electrodes close to the edge of the cap) or interpolated (other electrodes, not more than three electrodes per participant per sequence). Eye movements were corrected automatically (detection threshold 150 µV for horizontal, 250 µV for vertical movements, V 6.0, BESA, Ille et al., 2002). The data were epoched −100 to 975 ms around the phoneme pair onset and baseline-corrected at −100 to 0 ms. Remaining epochs with high-amplitude noise were omitted (automatic rejection level ±120 µV). Epochs were averaged together and subtraction curves were calculated by subtracting the averaged standard epochs from the averaged deviant epochs.

All participants had 80% or more accepted epochs in each sequence (average amounts in Ignore: 58, range = 57–60 in people with dyslexia and 59, range = 57–60 in control group; in AttendVowel: 29, range = 28–30 in people with dyslexia and 29, range = 27–30 in control group.) Due to the risk of motor artifacts when participants pressed the button to rule violations, MMN and P3a responses to rule violations in attentive condition were analyzed from the AttendVowel sequence instead of the AttendRule sequence (“target” condition).

### Quantification and Statistical Analysis of EEG Data

MMN and P3a peak amplitude latencies were defined from the subtraction curves at midline electrodes (Fz, FCz, and Cz) in the following time windows (from the second phoneme onset, that is, deviance onset): for MMN, 100 to 300 ms and, for P3a, 150 to 450 ms. As the latencies did not differ statistically significantly between groups or conditions (repeated-measures analysis of variance [RM-ANOVA] in both *p* > .05), the same time windows for people with dyslexia and controls and ignore and attentive conditions were used to calculate the MMN mean amplitudes (167–217 ms, centered on the average of peak latencies at the three midline electrodes in the control group in the ignore condition). This choice was made as the ignore condition in the control group was assumed to demonstrate the typical MMN (with minimal overlap of other, for example, attention-related components, particularly the N2b, see, for example, Näätänen et al., 1982). Because the components may still coexist in the subtraction curve, the response is hereinafter referred to as MMN/N2b. The P3a mean amplitudes were calculated from a latency window of 300 to 350 ms, centered on the mean peak latency at the three midline electrodes over groups and conditions.

Statistical significance of MMN/N2b and P3a responses was investigated with one-sample, two-tailed *t* tests at Fz (mean amplitude against 0, two responses, two groups, and two conditions equals eight *t* tests in total, Bonferroni-corrected for multiple comparisons). The effects of group and condition on the amplitudes of statistically significant responses were analyzed at nine fronto-central electrodes (F1, Fz, F2, FC1, FCz, FC2, C1, Cz, and C2) with an RM-ANOVA (between subjects factor: group; within-subjects factors: electrode, condition—effect of electrode not analyzed). Effect sizes are reported as partial eta-squared ( $\eta_{p}^{2}$ ).

#### Relationship to neuropsychological test performance

To study the relationship of MMN/N2b and P3a responses with reading-related skills, partial correlation analysis (with the effect of group partialed out) was conducted to the response amplitudes and relevant neuropsychological test scores (see Table 1). To reduce the amount of conducted tests, only MMN/N2b was included in the correlation analyses (as P3a demonstrated no statistically significant group differences; see the “Results” section). Furthermore, a mean amplitude was calculated for each response from a region of interest of nine fronto-central electrodes that were also used in the RM-ANOVAs.

Table 1 summarizes the relevant neuropsychological test scores. Tests related to verbal working memory (Digit span from Wechsler Memory Scale–Third Edition [WMS-III], Wechsler, 2008; Nonword span length, Laasonen et al., 2002), rapid naming (Rapid Alternating Stimulus naming RAS, Wolf, 1986), and phonological awareness (Pig Latin, Nevala et al., 2006) were included in the correlation analyses as these skills are associated with dyslexia (see, for example, Laasonen et al., 2009, 2010). Performance intelligence quotient ([PIQ] from Wechsler Adult Intelligence Scale–Third Edition [WAIS-III], Wechsler, 2005), verbal intelligence quotient (VIQ from WAIS-III, Wechsler, 2005), Word lists I and II, and Visual series (from WMS-III, Wechsler, 2008) are reported for the sake of completeness, but they were not included in correlation analyses. Results are reported both uncorrected and Bonferroni-corrected for multiple comparisons.

### Source Reconstruction and Statistical Analysis of Source Data

Source analyses were conducted post hoc using Brainstorm software (Tadel et al., 2011) for all group and condition effects that were significant at the sensor level. As individual magnetic resonance imagings (MRIs) were not available, ICBM152 anatomy was used (an unbiased average of 152 individuals, Fonov et al., 2009, 2011). On the basis of the ICBM152 anatomy, a three-layer boundary element model (BEM-models) with 1,082 vertices for the scalp and 642 for both the brain and the skull were computed, using openMEEG software package (Gramfort et al., 2010; Kybic et al., 2005). Brainstorm’s default conductivities for the scalp, skull, and the brain (relative conductivities: 1, 0.0125; or 1/80, and 1, respectively) were used. As no digitized electrode locations were available, default electrode scalp locations of the EEG cap layout (Biosemi Active two mk2 64-channel) were used.

For source analysis, noise covariance was estimated individually for each participant using pre-stimulus baselines for all stimulus averages recorded in all the sequences used in the experiment. Then, deviant-minus-standard subtraction curves were calculated for each participant and source activations for the responses in the latencies of interest (167–217 ms for the MMN, 300–350 ms for the P3a, as chosen for the sensor-level analyses) were computed, using weighted minimum norm estimate (Wmne) imaging (see, for example, Lin et al., 2006) method. As the study was conducted using EEG, unconstrained source orientations were used instead of limiting dipole orientation normal to the cortex. For noise covariance regularization, diagonal noise regularization with regularization parameter 1/λ = 3 was chosen. Further statistical analyses were conducted using strength of the dipoles at each BEM grid point (using pA m as unit), as is standard in Brainstorm software; figures depicting source activation also report dipole strength at each grid point, not current density (e.g., nA/mm^2^).

When differences between groups or conditions were statistically significant on sensor level, two-tailed permutation tests (paired for within-group comparisons, independent for between-group comparisons) with 1,000 randomizations were run for each BEM grid point on source level. As unconstrained source orientations were used, permutation analyses were conducted using absolute values for the sources. A *p* value of .05 was considered statistically significant. As statistical testing of the differences between the groups were conducted already in the sensor-level analyses and source analyses were conducted only as post hoc, no corrections for multiple comparisons were applied.

### Quantification and Statistical Analysis of Behavioral Data

Verbal responses of the participants were scored as 0 (*no/incorrect answer*), 1 (*partially correct answer*, for example, sound order, sound relationship, or “melody” changed), or 2 (*correct answer*, for example, sound pairs or groups of sounds had a falling instead of a rising pitch) after Ignore and Familiarization. Some individual participants reported hearing the /i/-/i/ pairs as two different phonemes and described the rule violation as an order change between the phonemes. They were given 2 points as this resulted in correct identification of the rule violation. Group differences were analyzed with nonparametric independent samples Mann–Whitney *U* test (distribution was skewed in both groups, see Table 3).

**Table 3.** The MMN/N2b and P3a Mean Amplitudes (µV) in Ignore and Attentive Conditions at Fz.

Measure	Condition	Group	Mean amplitude (*SD*)
MMN	Ignore	Dyslexia	−0.61 (0.80)**
		Control	−1.05 (0.90)***
MMN/N2b	Attentive	Dyslexia	−0.72 (0.88)**
		Control	−1.43 (1.26)***
P3a	Ignore	Dyslexia	0.57 (0.70)**
		Control	0.73 (0.60)***
	Attentive	Dyslexia	0.39 (1.36)
		Control	0.52 (0.94)*^a^

*Note*. Amplitudes differing statistically significantly from 0 are marked with asterisks. MMN = mismatch negativity.

a The attend-P3a in the controls did not survive Bonferroni correction, *p* < .006.

* *p* < .05. **p < .01. ***p < .001.

Accuracy (hit-ratio as percentage of hits per button presses) and speed (reaction times in ms) were calculated in the Familiarization and AttendRule sequences. The hit-ratio in Familiarization was calculated differently (as percentage of those hits per button presses that were not hits to the other, vowel deviant) as it required reacting to two deviants and it was not analyzed statistically. In the AttendRule sequence, one-sample *t* tests were conducted to test whether hit-ratios differed statistically significantly from chance-level (10%, probability of the rule violation in the sequence) and group differences in hit-ratios, and reaction times were studied with independent samples *t* tests or nonparametric independent samples Mann–Whitney *U* tests when distributions were not normal, reaction time: Shapiro–Wilk (27) = 0.905, *p* = .017.

## Results

### MMN/N2b and P3a: Group and Attention Effects

**Figure 2.**
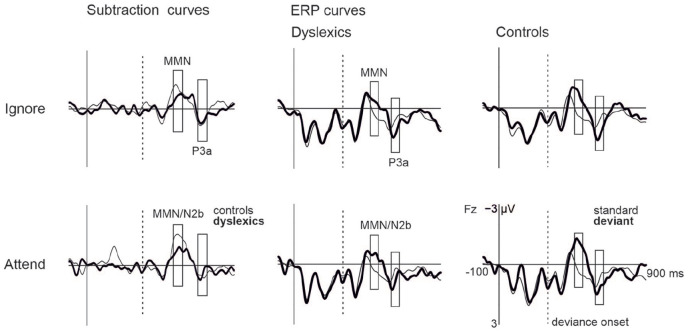
Subtraction curves (left) and ERP curves (right) depicting MMN/N2b and P3a responses in people with dyslexia and controls in the ignore and attentive conditions at Fz. *Note*. Bars illustrate the time windows where the mean amplitudes are calculated from, and the dashed line depicts the onset of the second phoneme (deviance onset).

**Figure 3.**
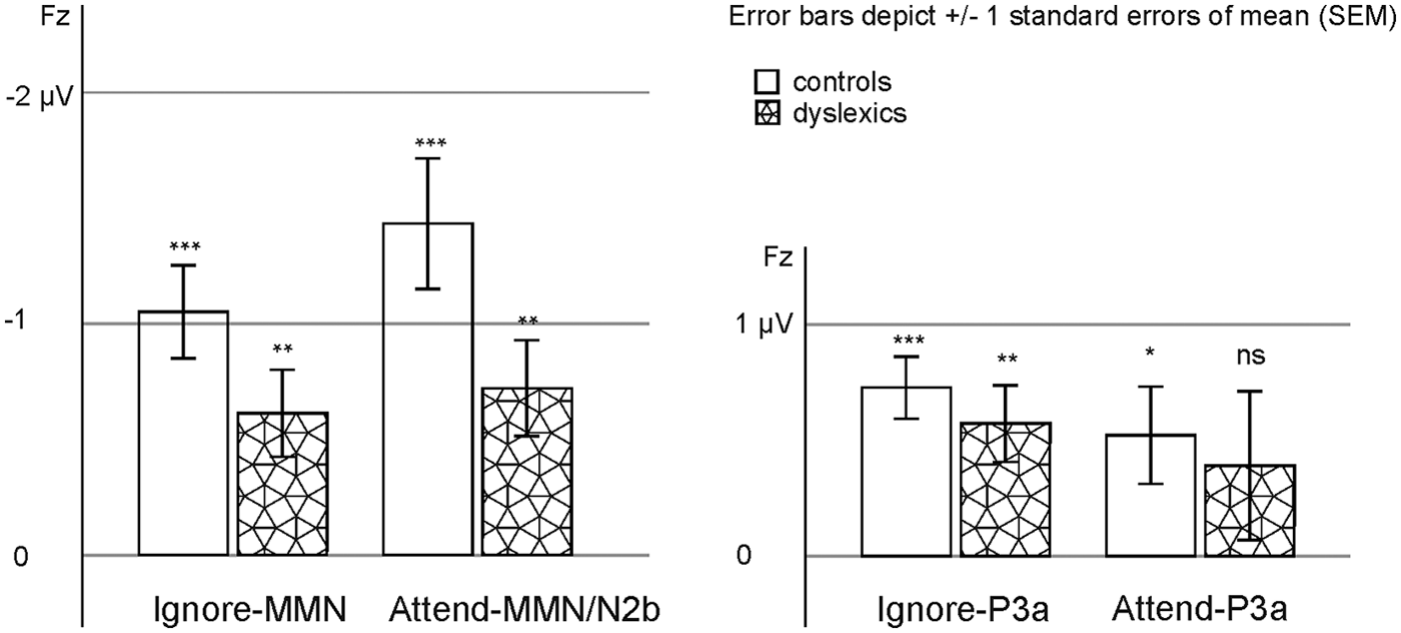
MMN/N2b and P3a mean amplitudes at Fz in controls and people with dyslexia in the ignore and attentive conditions. *Note*. Statistically significant *t*-test results are marked with asterisks, *p < .05. **p < .01. ***p < .001. Attend-P3a in controls was no longer significant after Bonferroni correction (criterion *p* < .006).

MMNs were statistically significant at Fz in both groups in ignore condition and MMN/N2bs in attentive condition (see Table 3, Figures 2 and 3, Supplemental Figure S3; the responses in the “target” condition are additionally illustrated in Supplemental Figure S4). The RM-ANOVA for group and condition effects showed a statistically significant group difference, indicating diminished MMN/N2bs in people with dyslexia across conditions, *F*(1, 36) = 4.239, *p* = .047, $\eta_{p}^{2}$ = .105, and a nonsignificant trend of larger MMN/N2b amplitudes in attentive compared with ignore condition, *F*(1, 36) = 3.044, *p* = .090, $\eta_{p}^{2}$ = .078. The interaction between group and condition was not statistically significant, *F*(1, 36) = 1.002, *p* = .324, $\eta_{p}^{2}$ = .027.

The P3a responses were statistically significant in the ignore but not in the attentive conditions in both groups (attend-P3a in controls did not survive correction for multiple comparisons; see Table 3) and therefore no further analyses were done for the P3a in the attentive condition. In RM-ANOVA for the P3a in the ignore condition, there was no statistically significant group effect, *F*(1, 36) = .471, *p* = .497, $\eta_{p}^{2}$ = .013.

As one participant in the dyslexia group and one control participant reported some explicit knowledge of the rule after the ignore condition (score >0), all analyses including the ignore condition were repeated with those participants excluded. The MMN and P3a responses remained statistically significant in the ignore condition in both groups (in all *p* < .006, Bonferroni-corrected criterion). The group difference in the RM-ANOVA for group and condition effects on MMN/N2b was nearly significant, *F*(1, 34) = 3.195, *p* = .083, $\eta_{p}^{2}$ = .086, whereas the condition effect, *F*(1, 34) = 2.764, *p* = .106, $\eta_{p}^{2}$ = .075, and the Group × Condition interaction, *F*(1, 34) = .599, *p* = .444, $\eta_{p}^{2}$ = .017, were not statistically significant.

### MMN/N2b Source Activations

In the ignore condition, the controls had stronger MMN source activation than people with dyslexia at the left hemisphere anterior temporal and inferior frontal regions (Supplemental Figure S1, critical *p* value = .05). In the attentive condition, the controls had stronger MMN/N2b source activation than people with dyslexia in the aforementioned areas and additionally in the right hemisphere, broadly distributed over the temporo-parietal regions (Supplemental Figure S2, critical *p* value = .05).

### Correlations With Reading Skills

**Figure 4.**
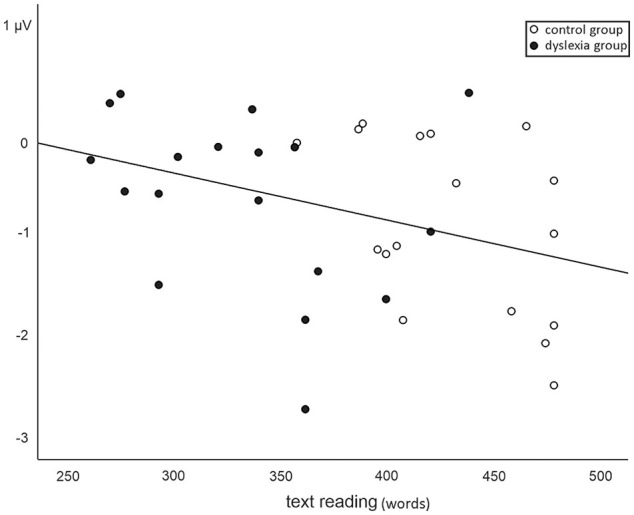
Relationship between MMN amplitude in the ignore condition and amount of correctly read words during text reading.

Partial correlation analysis revealed a moderate correlation between text reading and MMN amplitude in the ignore condition, *r* = −.363, *p* = .035, so that a greater amount of correctly read words within the time limit was associated with a larger MMN (see Figure 4; for complete statistics, see Supplemental Table S1). However, the correlation did not survive the Bonferroni-corrected criterion of *p* = .004.

### Behavioral Results

The majority of both people with dyslexia and control group participants were not able to describe the rule (violation) verbally after the Ignore or Familiarization sequences, with no statistically significant group differences (17 out of 18 people with dyslexia and 19 out of 20 control group participants had 0 points after the Ignore sequence, U = 180.50, *p* = 1.000; 11 out of 18 dyslexia group members and 13 out of 20 control group members had 0 points after Familiarization; U = 183.50, *p* = .919). Hit-rates were above chance-level in both groups in the AttendRule sequence—for control group, *t*(19) = 9.354, *p* < .001; for people with dyslexia, *t*(17) = 5.461, *p* < .001—and statistically significantly lower in people with dyslexia compared with the control group, 42.1 (*SD* = 25.9) versus 63.7 (*SD* = 25.7), *t*(36) = 2.617, *p* = .013. Hit-rates for people with dyslexia and controls in Familiarization were 29.7 (*SD* = 30.1) and 44.0 (*SD* = 36.4), respectively. Reaction times for people with dyslexia and controls in Familiarization were 667.1 (317.0) ms and 615.6 (285.3) ms, respectively. Reaction times for people with dyslexia and controls in AttendRule were 662.6 (179.2) ms and 622.8 (120.4) ms, respectively, with no statistically significant group differences, U = 189.000, *p* = .579.

## Discussion

This study determined whether neural and perceptual auditory rule extraction, which is vital for language functions and might reflect implicit learning problems, is abnormal in dyslexic adults. In line with our hypotheses, MMN/N2bs were diminished in people with dyslexia and they had lower hit-rates than control group participants in behavioral detection of the rule violations, suggesting impaired auditory rule extraction at neural and behavioral levels. Seemingly against the implicit learning deficit theory (Lum et al., 2013), the MMN/N2b group difference was seen across conditions, thus persisting in the attentive/explicit condition, and the hit-rates were lower in people with dyslexia than in the control group despite the provided explicit information of the rule. However, as discussed below, this result may still stem from an implicit learning deficit. Furthermore, the P3a was statistically significantly elicited only in the ignore/explicit condition, where no group differences were seen in its amplitude. Neural source estimations of the MMN/N2b revealed enhanced left anterior temporal and inferior frontal cortex activation in the control group compared with people with dyslexia across conditions, in line with our hypotheses, with additional group differences in the attentive condition in the right hemisphere. A tentative positive correlation was seen between the magnitude of the MMN in the ignore/implicit condition and reading performance, consistent with the suggested link between reading impairment and implicit learning problems (e.g., Pavlidou & Williams, 2014; Sperling et al., 2004).

Overall, the results suggest that the deficits in speech processing of people with dyslexia extend to processing of complex auditory rules at neural and perceptual levels. This may reflect challenges for people with dyslexia in learning perceptual categories, which is vital for learning the native language phonemes. Whether these challenges are seen only at the implicit level of processing or also in adopting or utilizing explicit information of auditory or speech stimulation may still call for further research.

### Auditory Rule Extraction, Implicit Processing, and Dyslexia

In this study, people with dyslexia had diminished MMN/N2bs to rule violations and poor performance in the behavioral detection of the rule violations, in line with our hypothesis on auditory rule extraction difficulties in dyslexia. Whereas some previous auditory ERP findings have shown deficient implicit rapid neural learning of novel word forms (Kimppa et al., 2018; see also Perrachione et al., 2016) and language-relevant rule processing (Bonte et al., 2007; Cantiani et al., 2013, 2015; Sabisch et al., 2006; see also Kujala et al., 2000) in dyslexia, this study specifically demonstrated both neural and behavioral deficits in dyslexia in the processing of a complex auditory regularity that had to be extracted during the experiment (and thus was not relevant for the participants’ native language).

We hypothesized that a group difference only in the ignore/implicit condition would offer neural evidence for the implicit learning deficit theory in dyslexia (e.g., Lum et al., 2013). However, the group difference persisted in the attentive/explicit condition, where people with dyslexia also demonstrated lowered hit-rates, challenging this interpretation of the results. As is evident from the hit-rates that stayed rather low throughout the present experiment, extracting the rule remained a challenging task for both groups also after the rule was explained to the participants. It is thus possible that, due to the task difficulty and the fast stimulus presentation rate, the participants actually could not extract explicit information from the stimuli, rendering the attentive/explicit condition rather implicit for them. This may be particularly true for the people with dyslexia due to their auditory and speech processing deficits. This view is also supported by the absence of a statistically significant P3a in the attentive/explicit condition (particularly in the dyslexic group), as P3a elicitation is more dependent on explicit awareness of the deviants than MMN elicitation (van Zuijen et al., 2006; Virtala et al., 2018).

The present results can thus be interpreted as consistent with the implicit learning theory of dyslexia, previously investigated using mainly behavioral methods (e.g., Gabay et al., 2015; Gabay & Holt, 2015; Lum et al., 2013). These studies found poorer implicit learning of stimulus regularities in visual, auditory, or motor sequences or in complex categories in dyslexia (review: Krishnan et al., 2016). General problems in implicit/procedural learning have been suggested to be a central cause for dyslexia (e.g., Pavlidou & Williams, 2014; Sperling et al., 2004). Gabay et al. (2015) specifically proposed that the phonological deficit in dyslexia could stem from a general impairment in implicit learning of *perceptual categories*. During early language development, this impairment would compromise the acquisition of the native language phoneme categories (Noordenbos & Serniclaes, 2015). Also in the present experiment, extracting the rule required categorization of the stimuli to rising versus falling pairs based on their invariant features, despite the continuous acoustic variation. Thus, the present findings support an impairment in forming perceptual categories in dyslexia at least in the auditory modality.

If deficient implicit rule extraction, possibly reflecting the broader difficulties in implicit learning of perceptual categories, is central for the development of dyslexia, the problems seen in this study should be evident early in life and associated with language and reading measures, particularly with categorical phoneme processing and its early development. Indeed, the present data showed a tentative association between faster reading speed and larger MMN amplitude in the ignore/implicit condition. While the result should be treated with caution due to the large amount of tests, it supports the above-described hypothesis that problems in implicit processing are related to problems in reading. The people with dyslexia in this study also demonstrated a deficit in processing of phoneme categories (Virtala et al., 2020), further supporting the view that a problem in forming auditory categories in dyslexia is evident in, but not restricted to, phoneme discrimination. These associations should be studied further by, for example, investigating whether infants at familial risk for dyslexia already demonstrate such deficits and whether they can predict language skills during early development. The MMN and the present auditory paradigm offer an ideal way to study this even in infancy, when behavioral tasks are challenging to administer.

### Dyslexia and Explicit Rule Extraction

In this study, all participants heard the stimuli first in an ignore condition and thereafter, prior to attentive conditions, they were given explicit information on the nature of the rule. The participants with dyslexia still displayed diminished MMN/N2b amplitudes across conditions. The group difference seemed to even grow numerically in the attentive/explicit compared with the ignore/implicit condition (although the Group × Condition interaction was not statistically significant; for mean amplitudes see Table 3). Source strengths in the right-hemispheric regions only showed group differences in the attentive/explicit condition but not in the ignore/implicit condition; however, these results need to be interpreted with caution (see the following). Participants with dyslexia also had lowered hit-ratios to the stimuli violating the rule, and their target-detection performance seemed to increase less (numerically) than that of the control group as a result of explicit information (29.7–42.1 in people with dyslexia vs. 44.0–63.7 in the control group between Familiarization and AttendRule sequences, although hit-ratios in the two phases are not fully comparable and were not statistically compared, see “Method” section).

Although these results may still reflect implicit processing deficits in people with dyslexia, as discussed above, an additional or alternative interpretation of the results is that the people with dyslexia had problems also in attentive/explicit processing of auditory rules. As people with dyslexia seemed to benefit less from the explicit information provided, it is possible that they had trouble in *making use* of this information. Their problems in rule extraction that persisted even after receiving explicit information of the rule suggests problems in declarative learning, seemingly against the proposed implicit learning deficit theory (Lum et al., 2013). However, when interpreting the present findings in relation to the implicit/procedural learning theory, it should be noted that this study investigated rule extraction in a speech sound context, where people with dyslexia can be expected to have deficits even at the explicit processing level (Giraud & Ramus, 2013; Schulte-Körne & Bruder, 2010; Vellutino et al., 2004). Consistent with this, also in a previous ERP study, people with dyslexia were poor in attentively detecting in which segment of a word or a tone pattern a duration change occurred (Kujala et al., 2006). This was evident as absent N2b responses and poor performance in a target-detection task in people with dyslexia.

It is also relevant to notice that the level of processing tapped by auditory ERPs differs markedly from the often-used behavioral paradigms of implicit learning (such as AGL and SRTT, Lum et al., 2013; Pothos, 2007), and therefore their findings may not be in unison if only some processing levels are impaired in dyslexia. More studies are needed to investigate how, for example, task demands and modality, which both differ between the present and previous implicit learning studies, affect the group differences obtained. Ideally, the implicit and explicit as well as neural and behavioral processing levels should be examined in the same study, as was attempted in the present experiment.

Surprisingly, the P3a response, which reflects an attention switch to stimulus changes (Wetzel & Schröger, 2014) was not statistically significantly elicited in either group in the attentive/explicit condition, whereas it was elicited in both groups in the ignore/implicit condition. Although the average amplitudes were smaller in the dyslexia group than in the control group across conditions (see Figures 2 and 3, Table 3), no statistically significant group difference was found in the ignore/implicit condition (while the group difference was not analyzed in the attentive/explicit condition). It should be noted that during the attentive MMN/N2b and P3a recording condition, the participants had to detect other, more salient deviants than the rule violations (vowel changes). Focusing on the task involving salient deviants may have caused damping down of implicit neural processing of the rule violations in both groups in the attentive condition, namely, the P3a can be reduced if the primary task is demanding or the deviant is not salient (see, for example, Harmony et al., 2000). Furthermore, this damping down may have been even more pronounced in the dyslexic group because the detection task was more difficult for the people with dyslexia than the control group and thus likely resulted in a higher cognitive load for them. This may also have contributed to the lack of an MMN enhancement or an N2b in the dyslexia group in the attentive/explicit condition, thus offering a possible alternative interpretation for the obtained group difference in that condition: perhaps the dyslexic group did not have problems with attentive/explicit rule extraction per se, but instead their neural processing of the rule was compromised by having to focus on the vowel changes.

### Rule Extraction and MMN Sources in Dyslexia

Besides identifying a neural marker of an auditory rule extraction deficit in dyslexia, this study sheds light on the neural structures that may underlie it. As illustrated in Supplemental Figures S1 and S2, the results showed diminished MMN source activation in people with dyslexia at the left anterior temporal and inferior frontal regions in both conditions and in the attentive condition, as well as in the right temporo-parietal region. Previously, implicit learning of novel word forms was demonstrated as an enhancement in the ERP waveform that originated from the left posterior middle temporal and anterior inferior frontal areas in typically reading adults (Kimppa et al., 2015), overlapping with areas that demonstrated group differences in the MMN sources in this study. As this enhancement was absent in children with dyslexia (Kimppa et al., 2018), the neural process may reflect problems in implicit learning for people with dyslexia (attributed to a failure to benefit from repetitions; see, for example, Ahissar, 2007; Perrachione et al., 2016).

The MMN source loci in this study were consistent with previous studies using language stimuli (for a review, see Paavilainen, 2013). The diminished activation in the left hemisphere of the dyslexia group is in line with that of, for example, Renvall and Hari (2003), who demonstrated diminished left-hemispheric MMNs in people with dyslexia in response to frequency changes (see also Kujala et al., 2003). While studies on the neurobiology of dyslexia have yielded mixed results, functional abnormalities have been observed in the left hemisphere in parietal, temporal, fusiform, and inferior frontal regions and structural abnormalities bilaterally in superior temporal areas (Richlan et al., 2009, 2013). Taken together, the neural sources demonstrating group differences in this study support the view that the neural deficits in dyslexia are most often seen in abnormalities in left-hemispheric activity.

In this study, source analysis was conducted to complement the sensor-level ERP analyses. As the setting was suboptimal (no individual MRIs, approximated electrode locations, and somewhat spare EEG electrode placement), results on source analyses should be cautiously interpreted as approximate. It is highly likely that the source localization error exceeds 1 cm or more (for discussion and quantification of these suboptimal settings on the accuracy of the forward models in source analyses, please see Acar & Makeig, 2013). However, as to the best of our knowledge, sources of neural activation (or abnormalities in source activation in comparison with controls) of the abstract-MMN have not thus far been studied in people with dyslexia, the present results can offer a valuable starting point for planning subsequent experiments on dyslexia and abstract-MMN.

## Conclusion

The present results highlight the broad nature of auditory processing difficulties in people with dyslexia. We demonstrated a rule extraction deficit in dyslexia using both behavioral and neural measures. We complemented the ERP analysis with source-level analyses that have been scarce in both abstract-MMN studies and in MMN studies on dyslexia.

The problems that people with dyslexia have in extraction of a complex auditory rule were seen across the ignore/implicit and attentive/explicit conditions. This is partly in line with the implicit learning deficit theory of dyslexia, supporting the view that deficient extraction of auditory categories may underlie the phonological deficits in dyslexia. After receiving explicit information on the rule, rule extraction problems of the people with dyslexia persisted in the attentive condition at both the neural and behavioral levels. As discussed above, rule violation detection may have been largely implicit even in the attentive condition. Alternatively, or additionally, this may reflect the difficulties that people with dyslexia have in adopting or utilizing the explicit information given to them during auditory or speech processing. Based on the present results, challenges in implicit extraction and effortless adoption of auditory rules may be central for language processing difficulties in dyslexia and related disorders.

## Supplemental Material

sj-docx-1-ldx-10.1177_0022219420988004 – Supplemental material for Impaired Perception and Neural Processing of Rules in Developmental Dyslexia Click here for additional data file. Supplemental material, sj-docx-1-ldx-10.1177_0022219420988004 for Impaired Perception and Neural Processing of Rules in Developmental Dyslexia by Paula Virtala, Eino Partanen and Teija Kujala in Journal of Learning Disabilities

sj-tif-1-ldx-10.1177_0022219420988004.tif – Supplemental material for Impaired Perception and Neural Processing of Rules in Developmental Dyslexia Click here for additional data file. Supplemental material, sj-tif-1-ldx-10.1177_0022219420988004.tif for Impaired Perception and Neural Processing of Rules in Developmental Dyslexia by Paula Virtala, Eino Partanen and Teija Kujala in Journal of Learning Disabilities

sj-tif-2-ldx-10.1177_0022219420988004 – Supplemental material for Impaired Perception and Neural Processing of Rules in Developmental Dyslexia Click here for additional data file. Supplemental material, sj-tif-2-ldx-10.1177_0022219420988004 for Impaired Perception and Neural Processing of Rules in Developmental Dyslexia by Paula Virtala, Eino Partanen and Teija Kujala in Journal of Learning Disabilities

sj-tif-3-ldx-10.1177_0022219420988004 – Supplemental material for Impaired Perception and Neural Processing of Rules in Developmental Dyslexia Click here for additional data file. Supplemental material, sj-tif-3-ldx-10.1177_0022219420988004 for Impaired Perception and Neural Processing of Rules in Developmental Dyslexia by Paula Virtala, Eino Partanen and Teija Kujala in Journal of Learning Disabilities

sj-tif-4-ldx-10.1177_0022219420988004 – Supplemental material for Impaired Perception and Neural Processing of Rules in Developmental Dyslexia Click here for additional data file. Supplemental material, sj-tif-4-ldx-10.1177_0022219420988004 for Impaired Perception and Neural Processing of Rules in Developmental Dyslexia by Paula Virtala, Eino Partanen and Teija Kujala in Journal of Learning Disabilities
