# Supplementary material for: Impaired Perception and Neural Processing of Rules in Developmental Dyslexia
Source: J Learn Disabil. 2021 Jan 21;54(6):452–65. doi: 10.1177/0022219420988004 (PMC8559173; doi:10.1177/0022219420988004)
Supplement: sj-docx-1-ldx-10.1177_0022219420988004 – Supplemental material for Impaired Perception and Neural Processing of Rules in Developmental Dyslexia [file sj-docx-1-ldx-10.1177_0022219420988004.docx]

| **JOURNAL OF LEARNING DISABILITIES SUPPLEMENTAL FILE**  **Table S1.** *Partial correlations (effect of group partialed out) of MMN and MMN/N2b mean amplitudes on a ROI of nine fronto-central electrodes with reading-related subtests.*  **ARTICLE TITLE: Impaired perception and neural processing of rules in developmental dyslexia** | | |
| --- | --- | --- |
| Subtest | MMN, ignore | MMN/N2b, attentive |
| Digit span (WMS-III, Wechsler, 2008) | 0,11 | 0,18 |
| Nonword span, length (Laasonen et al., 2002) | -0,19 | -0,13 |
| Pig Latin (Nevala et al., 2006) | 0,31 | -0,11 |
| RAS, speed in 2nd trial (Wolf, 1986) | 0,07 | -0,01 |
| Word list reading, time (Nevala et al., 2006) | 0,20 | -0,19 |
| Nonword list reading, time (Nevala et al., 2006) | 0,25 | -0,14 |
| Text reading, amount of words (Nevala et al., 2006) | -0,36* | -0,04 |
| * p < .05 |  |  |
